# SPARC promoter hypermethylation in colorectal cancers can be reversed by 5-Aza-2′deoxycytidine to increase SPARC expression and improve therapy response

**DOI:** 10.1038/sj.bjc.6604377

**Published:** 2008-05-06

**Authors:** S Cheetham, M J Tang, F Mesak, H Kennecke, D Owen, I T Tai

**Affiliations:** 1 Division of Gastroenterology, University of British Columbia and Genome Sciences Centre, British Columbia Cancer Agency, Vancouver, British Columbia, Canada; 2 Division of Medical Oncology, British Columbia Cancer Agency, Vancouver, British Columbia, Canada; 3 Division of Pathology, University of British Columbia, Vancouver, British Columbia, Canada

**Keywords:** SPARC, methylation, 5-Aza-2′deoxycytidine, colorectal cancer

## Abstract

Poor clinical outcomes in cancer can often be attributed to inadequate response to chemotherapy. Strategies to overcome either primary or acquired chemoresistance may ultimately impact on patients' survival favourably. We previously showed that lower levels of SPARC were associated with therapy-refractory colorectal cancers (CRC), and that upregulating its expression enhances chemo-sensitivity resulting in greater tumour regression *in vivo*. Here, we examined aberrant hypermethylation of the *SPARC* promoter as a potential mechanism for repressing *SPARC* in CRCs and whether restoration of its expression with a demethylating agent 5-Aza-2′deoxycytidine (5-Aza) could enhance chemosensitivity. Initially, the methylation status of the *SPARC* promoter from primary human CRCs were assessed following isolation of genomic DNA from laser capture microdissected specimens by direct DNA sequencing. MIP101, RKO, HCT 116, and HT-29 CRC cell lines were also used to evaluate the effect of 5-Aza on: *SPARC* promoter methylation, *SPARC* expression, the interaction between DNMT1 and the *SPARC* promoter (ChIP assay), cell viability, apoptosis, and cell proliferation. Our results revealed global hypermethylation of the *SPARC* promoter in CRCs, and identified specific CpG sites that were consistently methylated in CRCs but not in normal colon. We also demonstrate that *SPARC* repression in CRC cell lines could be reversed following exposure to 5-Aza, which resulted in increased *SPARC* expression, leading to a significant reduction in cell viability (by an additional 39% in RKO cells) and greater apoptosis (an additional 18% in RKO cells), when combined with 5-FU *in vitro* (in comparison to 5-FU alone). Our exciting findings suggest potential diagnostic markers of CRCs based on specific methylated CpG sites. Moreover, the results reveal the therapeutic utility of employing demethylating agents to improve response through augmentation of SPARC expression.

The molecular pathogenesis associated with colorectal cancer (CRC), as the second leading cause of cancer death in North America, is the focus of extensive investigation. Many of the genetic changes leading to the multi-step progression from adenoma to carcinoma have been described, such as mutations of the adenomatous polyposis coli (APC) gene, K-Ras (Morris *et al*, 1996), SMAD2, SMAD4 (Thiagalingam *et al*, 1996), p53 (Nigro *et al*, 1989), and mismatch repair genes (hMSH2, hMLH1, PMS1, GTBP (Kinzler and Vogelstein, 1996; Perucho, 1996; Chung, 2000). Hypermethylation of various genes have also been implicated in colorectal cancer development and progression (Toyota *et al*, 1999). Examples include several tumour suppressor genes, such as INK4A(p16) cell cycle regulator (Herman *et al*, 1995), as well as others, such as hMLH1 nucleoside mismatch repair gene (Kane *et al*, 1997), THBS1 angiogenesis inhibitor (Ahuja *et al*, 1997), and TIMP3 metastasis suppressor (Cameron *et al*, 1999).

Secreted protein acidic and rich in cysteine (SPARC), also known as osteonectin, is a matricellular protein involved in wound repair, cell migration and differentiation (Sage *et al*, 1989; Yan and Sage, 1999; Bradshaw and Sage, 2001; Brekken and Sage, 2001). The expression of SPARC is variable in different cancers, and its role in tumorigenesis appears complex and not well defined (Wewer *et al*, 1988; Bellahcene and Castronovo, 1995; Ledda *et al*, 1997; Said and Motamed, 2005; Tai *et al*, 2005; DiMartino *et al*, 2006; Rodriguez-Jimenez *et al*, 2007). For example, in neuroblastoma SPARC impairs tumour growth (Chlenski *et al*, 2006), while in another type of brain cancer, glioblastoma, SPARC induces metastasis and invasion (Rich *et al*, 2003). In some tissues, it appears to have tumour suppressing properties by conferring growth inhibition to cancers of the ovaries (Yiu *et al*, 2001), lung (Brekken *et al*, 2003), breast (Dhanesuan *et al*, 2002), neuroblastomas (Chlenski *et al*, 2002), and leukaemia (DiMartino *et al*, 2006). In addition, absence of SPARC in SPARC −/− mice promotes the growth of pancreatic and lung cancers (Brekken *et al*, 2003; Puolakkainen *et al*, 2004), thereby demonstrating that SPARC is capable of suppressing tumour growth in certain cancers due to endogenous expression of, or as a result of exogenous exposure to SPARC. Our laboratory recently correlated low levels of SPARC expression in colorectal cancers with decreased sensitivity to chemotherapy, and showed that reversal of therapy resistance could be achieved by upregulating SPARC expression or exogenous exposure to higher levels of SPARC either *in vitro* or *in vivo* (Tai *et al*, 2005). This change in SPARC expression in tumorigenesis and its role in promoting chemotherapy sensitivity led us to investigate the potential mechanisms involved in repressing *SPARC* in colorectal cancers. Since aberrant methylation appears to play a role in CRC, and *SPARC* promoter hypermethylation appears to be responsible for its low expression in cancers of the pancreas (Sato *et al*, 2003), lung (Suzuki *et al*, 2005), and leukaemia (DiMartino *et al*, 2006), we decided to evaluate the possibility that: (1) this mechanism may also be involved in suppressing *SPARC* in CRCs; and (2) whether a demethylating agent could reverse the methylated state of the *SPARC* promoter in CRC, resulting in higher SPARC expression and greater sensitivity of tumours to chemotherapy.

## MATERIALS AND METHODS

### Clinical samples, cell lines and DNA extraction

**Figure 1:**
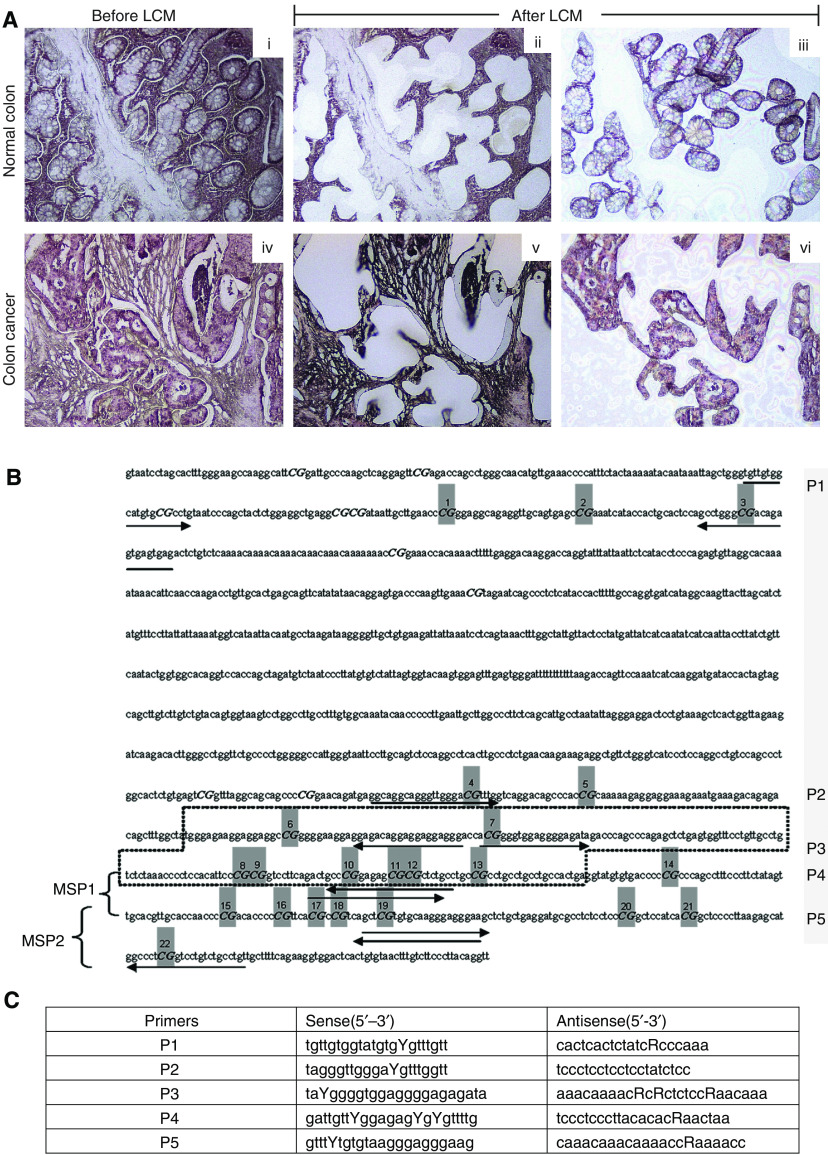
Histological images of specimens of normal colon or colon cancer before (**A**-i, iv) and after (**A**-ii, iii, v, vi) laser capture microdissection (LCM); only colonic epithelium represented by images (iii) or (vi) were used for analysis. (**B**) Map of the CpG island in the promoter region at 5′ of the *SPARC* gene, including regions flanked by the bisulfite sequencing primers (P1–P5, arrows and sequences), numbered CG positions within the sequencing region (grey), exon1 (boxed). Brackets show regions amplified using methylation-specific primers MSP1 and MSP2. (**C**) Five sets of primers were used to sequence the regions shown in (**B**).

Archival paraffin-embedded tissues of CRC and normal colon were used to determine the methylation status of the *SPARC* promoter (Table 1). All specimens of normal colon were identified from individuals who had undergone surgery for diverticular disease of the colon. This study was conducted with permission from our Institutional Ethics Review Board. From each specimen, genomic DNA was extracted from the colonic epithelia that were identified and captured by laser capture microdissection (Figure 1A) (Molecular Machine & Industries, *μ*Cut Laser Microdissection).

Human colorectal cancer cell lines, MIP 101 (Tai *et al*, 2005), RKO, HCT116 and HT29 (ATCC), and a normal colon cell line CCD-112CoN (ATCC), were maintained in DMEM supplemented with 10% fetal bovine serum; 1% Kanamycin, Streptomycin-Penicillin, and incubated at 37°C and 5% CO_2_. To determine the effect of 5-Aza-2′deoxycytidine (5-Aza, demethylating agent) on SPARC promoter methylation, 4 *μ*M of 5-Aza was added to the culture media (Suzuki *et al*, 2005) for a period of 7 days with media changes on day 4, before assessing its effect on cell viability (WST-1), apoptosis (Caspase 3/7 and TUNEL), cell proliferation, and *SPARC* promoter methylation (by bisulfite sequencing).

For DNA extraction, microdissected clinical specimens or cells harvested from CRC cell lines were digested overnight at 42°C (digestion buffer: 10 mM Tris-HCl [pH8], 1 mM EDTA, 1% Tween 20, 0.5% Proteinase K) then heated to 95°C for 10 min. One volume of phenol/chloroform/isoamyl alcohol (25 : 24 : 1) was mixed by inversion with the digested samples and added to the Phase Lock Gel™ Eppendorf tubes. After spinning at 14 000 × **g** for 5 min at room temperature, the aqueous layer was removed to which 1 *μ*l of glycogen (20 mg/ml) was added. Precipitation of the DNA was performed with three volumes of 95% ethanol, 0.1 M NaOAc for 2 h at −20°C and then centrifuged at 14 000 × **g** for 10 min at room temperature. DNA pellets were washed with 70% ethanol, spun at 14 000 × **g** for 10 min, air dried and resuspended in 10 *μ*l of modified TE (10 mM Tris (pH 8)/0.1 mM EDTA).

### DNA bisulfite sequencing, methylation-specific PCR, and RT-PCR

Direct DNA sequencing was performed after bisulfite treatment (Herman *et al*, 1996), using Methylamp One-step modification kit (Epigentek) following the manufacturer's instructions, with primers covering a CpG region 1500 bp upstream and including exon1 of *SPARC* (Figure 1B and C).

Methylation-specific PCR (MSP) was also performed after bisulfite modification, using methylation-specific primers, which were designed to recognise bisulfite-induced modifications of unmethylated cytosines. Two primer sets, MSP1 and MSP2, were used to target the CpG island located in the putative promoter region of *SPARC* (Sato *et al*, 2003; Wang *et al*, 2005). Primers targeting INK4A(p16), a gene found to be hypermethylated in CRC, were used as control (Mund *et al*, 2005).

For RT-PCR, mRNA isolated from CRC cell lines were extracted using Trizol (Invitrogen, Burlington, Ontario, Canada). Primers used to amplify SPARC and PCR conditions were previously described (Tai *et al*, 2005). Amplicons were separated in 2.5% agarose gel electrophoresis.

### Chromatin immunoprecipitation assay

To evaluate the effect of 5-Aza exposure on the interaction between Dnmt1 (a DNA methyltransferase) with the promoter region of *SPARC*, a chromatin immunoprecipitation (ChIP) assay was performed using a ChIP kit (Epigentek) as per the manufacturer's instructions. Cells were fixed with formaldehyde for protein/DNA crosslinking and lysed. The DNA was sheared by sonication (10 pulses, 30 sec on 30 sec off) and added to a well coated with the antibody to the protein of interest (anti-Dnmt1, cat no. A-1001, Epigentek). Washes were performed to remove unbound material, while Dnmt1-bound DNA was released by protein digestion with proteinase K. The DNA was purified through a column and PCR was performed using primers designed to target the *SPARC* promoter region spanning the site of interaction with Dnmt1 (Rhee *et al*, 2000), sense 5′-CCCCGGAACAGATGAGGCA-3′, anti-sense 5′-AGGGGAGCCGTGATGGAGCC-3′ (annealing 58°C), while INK4A(p16) was used as control (Lin *et al*, 2007). The sole modification to the manufacturer's protocol was the blocking of the antibody-coated well with 1% skim milk for 1 h to reduce background.

### Cell viability, proliferation and clonogenic assay

Cell viability was evaluated using WST-1 (Roche) following the manufacturer's instructions. Cells were seeded at 50% confluence per well in triplicate into a 96-well plate and exposed 24 h later to 4 *μ*M 5-Aza for a total of 5 days, with the addition of 1000 *μ*M of 5-FU in the last 24 h prior to WST assay. To evaluate the specificity of the effects of 5-Aza on *SPARC* promoter methylation, we also assessed the effect of knocking-down SPARC expression using SPARC siRNA. Cells seeded in triplicate wells in 96-well plates were transiently transfected 24 h later with SPARC siRNA designed to target sequences 5′-AATTCGAGAAGAACTATAACA-3′ and 5′-AATGTGCAAGCTGGATGAGAA-3′ (Qiagen, Missassauga, Ontario, Canada). HiPerfect Reagent (Qiagen) was used for the transient transfection. Scramble oligonucletide sequences were used as non-silencing controls for these studies. In all 4 *μ*M 5-Aza was added into the wells the same day as the siRNA transfection and incubated for a total of 5 days. Again, cells were also exposed to 1000 *μ*M 5-FU in the last 24 h prior to WST assay. Three independent replicates were analyzed, with results represented as mean±s.e.

To determine the rate of cell proliferation, cells exposed to 5-Aza for 7 days (and untreated controls) were seeded at 20 000 cells per well into a 48-well plate. Cells were counted from duplicate wells at 24, 48 and 72 h. The effect of a 24-h 5-FU exposure on cell proliferation, in the presence or absence of 5-Aza, was also determined. Results were based on two independent experiments.

For the clonogenic assay, 5-Aza treated and untreated control cells were seeded at 1000 cells per well in a 48-well plate. Twenty-four hours later, cells were incubated with 100–1000 *μ*M 5-FU for 7 days, with a change in media and drug on day 4. Cells were then stained with 0.2% crystal violet. The number of colonies in the treated group was calculated based on the colonies formed from the control, untreated cells. Assays were performed in triplicate.

### Apoptosis

Cells that were exposed to 4 *μ*M 5-Aza (and untreated controls) were seeded into a 48-well plate at 100 000 cells per well. Twelve hours later, cells were incubated with 1000 *μ*M of 5-FU for an additional 24 h before harvesting the cells for TUNEL assays. To assess the effect of 5-Aza in the absence of SPARC expression, we also transiently transfected cells with SPARC siRNA 24-h after seeding (as described earlier), in combination with 4 *μ*M 5-Aza for a total of 5 days. In the last 24 h of incubation with 5-Aza, cells were also exposed to 1000 *μ*M 5-FU prior to harvesting, and subsequently used for caspase 3/7 assays. Specifically, 20 *μ*g of total protein was isolated from cell lysates, and using a 1 : 1 dilution of Caspase Glo-3/7 Substrate (Promega, Madison, WI, USA), relative luminescence units (RLU) were quantified using Viktor 1420 Multilabel counter (Perkin Elmer), as previously described (Taghizadeh *et al*, 2007; Tang and Tai, 2007). For TUNEL assay, suspension and attached cells were collected and fixed onto glass slides with Shandon cytospin at 2000 r.p.m. for 10 min, and stained using the DeadEnd II Fluorometric TUNEL System (Promega). The number of TUNEL-positive cells was counted and averaged from four different fields, with slides read independently by different individuals in a blinded fashion. Triplicate assays were performed.

### Immunoblotting

Cell lysates from MIP 101 cells incubated with 4 *μ*M of 5-Aza for 7 days were collected and processed as previously described (Tai *et al*, 2005; Taghizadeh *et al*, 2007; Tang and Tai, 2007). Antibodies to SPARC (1 *μ*g/ml; Haematologic Technologies Inc.) and tubulin (0.2 *μ*g/ml; Sigma-Aldrich) were used.

### Statistics

Statistical difference between experimental groups were analyzed using Student's *t*-test, with significance defined as *P*<0.05, by Smith's Statistical package.

## RESULTS

### Hypermethylation of the *SPARC* promoter was more commonly observed in human colorectal cancers than in normal colon

**Figure 2:**
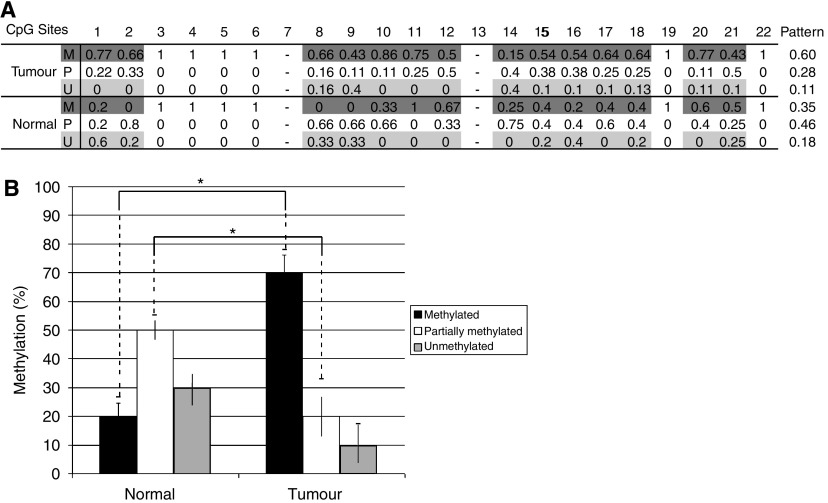
Direct bisulfite DNA sequencing of human colorectal cancers and normal colon in the 1500 bp region 5′ of the SPARC gene (including exon1-intron1) (**A**) The frequency of M=methylation, P=partial methylation, and U=unmethylation at each CpG site (numbers correspond to grey boxes in Figure 1) is provided for colorectal cancers (tumour) and normal colon (normal). The pattern of methylation considers only the frequency of methylation within CpG sites with variable status (thus excluding the consistently methylated CpG sites in CRC and controls). (**B**) Percentage of methylated CpG sites in the SPARC promotor region in CRC or normal colon samples when considering positions 1–2, 8–10, 16–18 only. Significance was determined *P*<0.05.

To determine more definitively the methylation status of the CpG sites within the 1500 bp region upstream of the *SPARC* promoter and including exon 1, bisulfite-treated DNA from microdissected clinical specimens were sequenced (Herman *et al*, 1996). Overall, a significantly higher proportion of CpG sites were methylated within the *SPARC* promoter in colorectal cancers (60%), while only 35% of the sites were methylated in normal colon (*P*=0.03) (Figure 2A). The proportion of partially methylated sites were significantly lower in the colorectal cancers (28%) compared to the normal colon (46%) (*P*=0.04). In the normal colon, there appeared to be a similar frequency of CpG sites that were either completely methylated, partially or completely unmethylated. We also noted that CpG positions 1–2, 8–10, 16–18 were more frequently methylated in colorectal cancer, in comparison to normal colon (Figure 2B).

In addition, when we examined the number of colorectal cancers with greater than 50% methylated CpG sites in the 1500 bp region sequenced, 80% of colorectal cancer (8 of 10 samples) met this criteria, while only 20% of normal colons (one of five samples) could be considered as hypermethylated. An interesting observation was the difference in overall survival between the two patients with Stage IV colorectal cancer: one patient with hypermethylation in 95% of the CpG sites examined survived 18 months, while the other one with hypermethylation in only 60% of the sites survived 37 months. The same patient with Stage IV disease and hypermethylation in 95% of CpG sites within the *SPARC* promoter from samples isolated from the tumour also had an area of the surrounding normal colon examined, and this showed hypermethylation in only 50% of the CpG sites. Given the differential degree of methylation of the *SPARC* promoter between the tumour and normal colon within the same individual, this suggests that the degree of hypermethylation in the *SPARC* promoter within the tumour is likely related to the diseased state.

### Hypermethylation of the promoter region of SPARC was observed in colorectal cancer cell lines

**Figure 3:**
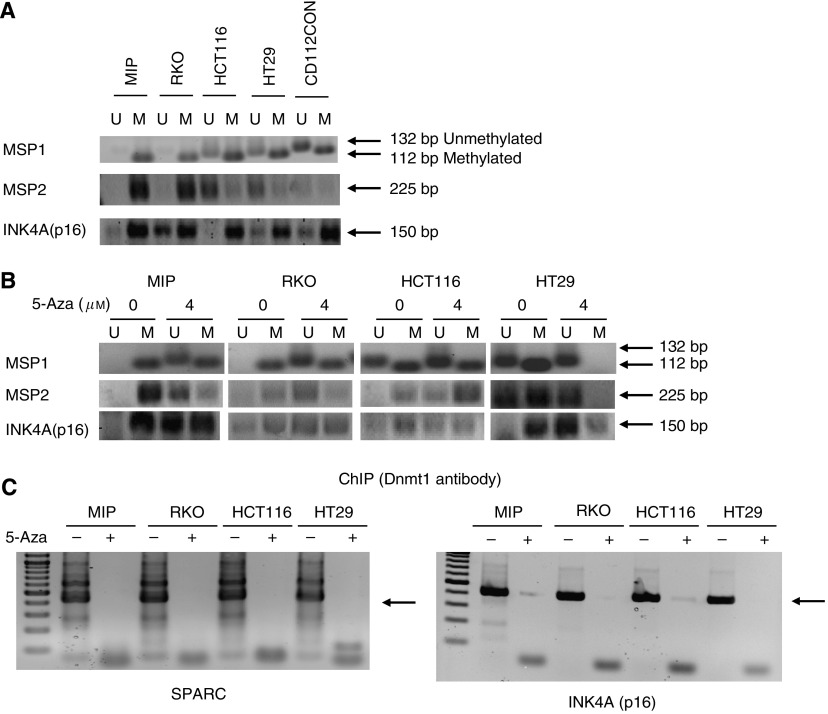
Methylation-specific PCR (MSP) and ChIP assays of CRC cells. Primers targeting the promoter-exon 1 region of *SPARC* were used to determine its methylation status in: (**A**) CRC cell lines (MIP101, RKO, HCT116, HT29) and normal colon (CCD-112CoN); and (**B**) CRC cell lines after exposure to 5-Aza for 7 days. Hypermethylation (MIP101, RKO) as well as hemimethylation (HCT116, HT29) of the promoter region of *SPARC* was observed, which resulted in unmethylation of the *SPARC* promoter after exposure to 5-Aza. (**C**) ChIP assay showing amplified PCR products of the regions within the *SPARC* and *INK4A*(*p16*) promoters that interact with Dnmt1 in CRC cell lines not exposed to 5-Aza.

We assessed the methylation status of four colorectal cancer cell lines (MIP101, RKO, HT29, and HCT 116) and one normal colon cell line (CCD 112CoN) and observed hypermethylation in the promoter region of MIP 101 and RKO cells flanked by both MSP1 and MSP2 (Figure 3A). In HCT116 and HT29 cells, partial methylation was noted in both cell lines with methylated CpG sites in the MSP1 region but unmethylated in the MSP2 region (Figure 3A). Normal colon cell line CCD-112CoN also showed partial methylation, with a methylated MSP1 region and unmethylated MSP2 region (Figure 3A). In addition, we also assessed the methylation status of the promoter of the *INK4A*(*p16*) gene, which is hypermethylated in colorectal cancer, and noted hypermethylation in MIP101 and HCT 116 cells, and partial methylation in RKO and HT 29 CRC cells. Partial methylation was also observed in the promoter region of this gene in normal colon cell line CCD-112CoN.

Exposure to a demethylating agent, 5-Aza (4 *μ*M), for 7 days *in vitro* resulted in a change in the methylation status in the MSP1 and MSP2 regions of the promoters for both *SPARC* and *INK4A*(*p16*) in all four CRC cell lines (Figure 3B). Unmethylated regions were now detected after incubation with 5-Aza, especially in those cell lines (MIP 101 and RKO cells) where there was complete methylation in both MSP regions. Exposure of HT29 cells to 5-Aza changed the *SPARC* promoter from a hemimethylated to a completely unmethylated state, while there appeared to be no significant effect of this demethylating agent on HCT 116 cells, which remained hemimethylated. Reversal of hypermethylation within the region of the *SPARC* promoter by 5-Aza was confirmed by ChIP assays. In all CRC cell lines, an interaction between the *SPARC* promoter with Dnmt1 (a DNA methyltransferase that catalyses the transfer of a methyl group to DNA, resulting in DNA methylation) could be detected (Figure 3C). However, following exposure to 5-Aza, this interaction was no longer observed.

### Exposure to demethylating agent, 5-Aza-2′deoxycytidine, increases the sensitivity to chemotherapy in colorectal cancer cells with methylated *SPARC* promoter

**Figure 4:**
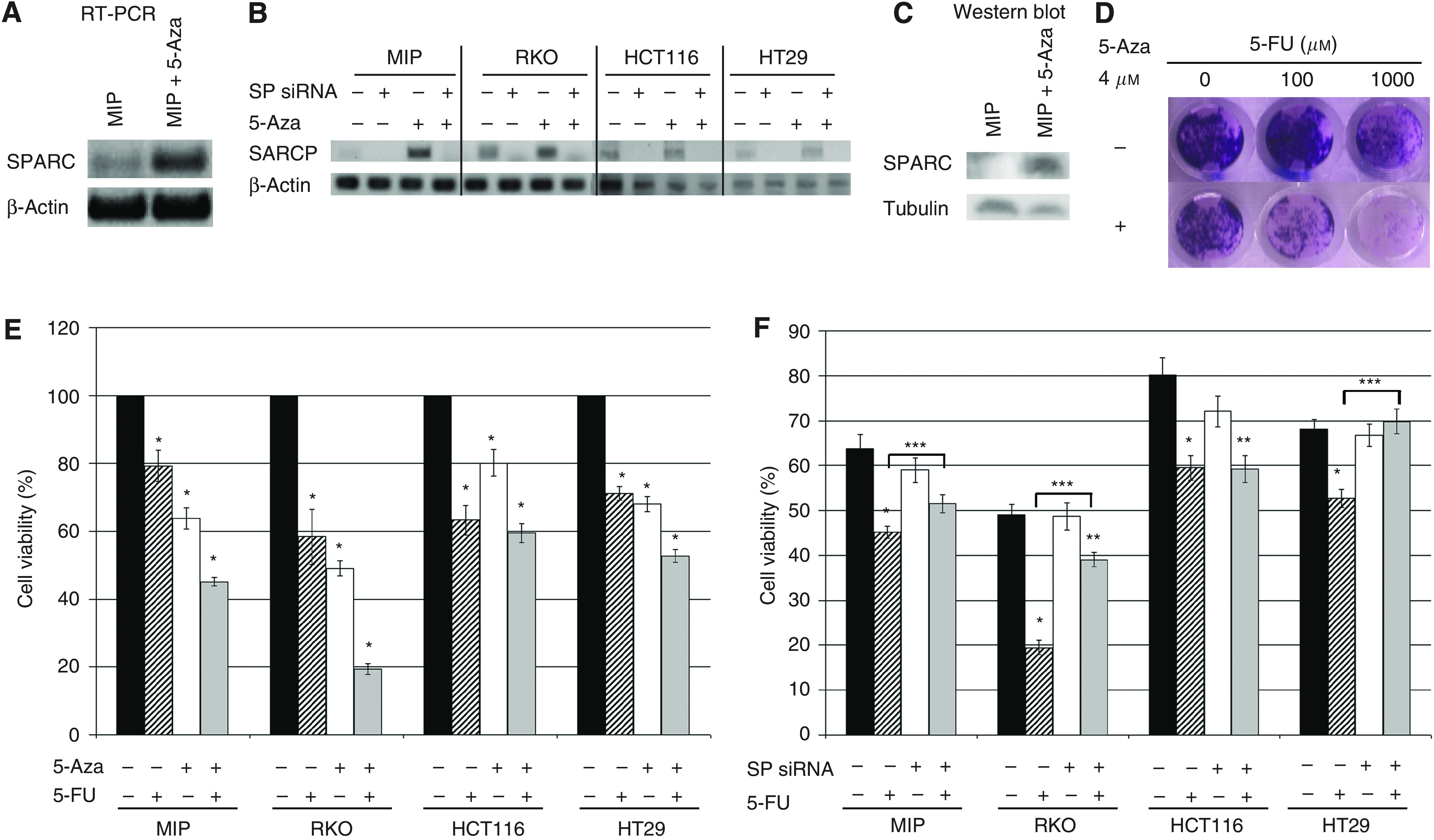
The effect of incubating CRC cells to 5-Aza, a demethylating agent, prior to exposure to 5-FU. Changes in SPARC expression following incubation with 5-Aza was evaluated by: (**A** and **B**) RT-PCR and (**C**) Immunoblot. (**D**) Clonogenic assay of MIP101 cells following preincubation with 5-Aza and incremental concentrations of 5-FU showed enhanced sensitivity in cells pre-incubated 5-Aza. (**E**) Cell viability was assessed following a 4-day preincubation with 4 *μ*M 5-Aza and 24-h exposure to 5-FU; and (**F**) in the presence/absence of SPARC siRNA transfection (**F**, note that all experimental groups were exposed to 5-Aza). (**E**) Increased sensitivity to 1000 *μ*M 5-FU was observed following preincubation with 5-Aza in MIP101, RKO and HT29 cells (‘^*^’ *P*<0.05, when compared with control, untreated cells). Cell viability increased despite pre-exposure to 5-Aza and treatment with 5-FU in cells transfected with SPARC siRNA (**F**), (*P*<0.05 for: ‘^*^’ compared with control (SPARC siRNA(−)/5-FU(−) treated cells; ‘^**^’ compared with SPARC siRNA(+)/5-FU(−) cells; and ‘^***^’ comparison between SPARC siRNA(−)/5-FU(+) and SPARC siRNA(+)/5-FU(+)). Results represented as mean±s.e.

We previously demonstrated that higher levels of SPARC expression was associated with greater sensitivity to chemotherapy (Tai *et al*, 2005). As noted above, hypermethylation of the *SPARC* promoter could be reversed following incubation with 5-Aza (Figure 3B), which also resulted in higher levels of *SPARC* gene (Figure 4A and B) and protein (Figure 4C) expression. Therefore, we next examined if pre-incubation with 5-Aza would influence the sensitivity of MIP101, RKO, HCT116 and HT29 CRC cells to 5-FU. Results of clonogenic assays revealed fewer colonies in the MIP101 cells exposed to both 5-Aza and 5-FU, in comparison to exposure to 5-FU alone (Figure 4D). These results correlate with the next set of observations demonstrating a significant reduction in cell viability in cells pre-incubated with 5-Aza before exposure to 5-FU (Figure 4E). In MIP101 cells, pre-incubation with 5-Aza resulted in a significant decrease in cell viability when cells were subsequently treated with 1000 *μ*M of 5-FU in comparison to 5-FU alone: from 79.2±4.6% viable cells (5-FU only) to 45.1±1.3 (*P*=0.000002). A similar reduction was observed with RKO (from 58.4±8.1% viable cells, 5-FU alone; to 19.4%±1.6, combination of 5-Aza and 5-FU, *P*=0.0008) and HT29 (from 71.1±2.0% viable cells, 5-FU; to 52.7±2.0%, combination 5-Aza and 5-FU, *P*=0.00005) cells (Figure 4E). HCT116 was the only cell line that did not respond significantly to pre-incubation with 5-Aza.

To determine if the reduction in cell viability following incubation with 5-Aza was occurring predominantly through its effect on SPARC, we proceeded to knock-down SPARC expression in our four CRC cell lines that were also exposed to 5-Aza and later, 5-FU. Our results show that in the absence of SPARC, the effect of 5-Aza in further reducing cell viability following exposure to 5-FU could be abolished (Figure 4F). In MIP101 cells exposed to 5-Aza and 5-FU, cell viability increased from 45.2±1.3% (scramble control) to 51.5±2.0% (SPARC siRNA) (*P*=0.02). Similar results were seen in RKO (increased from 19.4±1.6% to 39.0±1.6%, *P*=0.000006), and HT29 (increased from 52.7±1.9% to 69.8±2.8%, *P*=0.0005). Only the response of HCT 116 cells to SPARC siRNA remained unchanged. These observations indicate that the ability of 5-Aza to improve sensitivity of cells to 5-FU occurs, in part, by increasing *SPARC* gene expression following demethylation of its promoter by 5-Aza.

**Figure 5:**
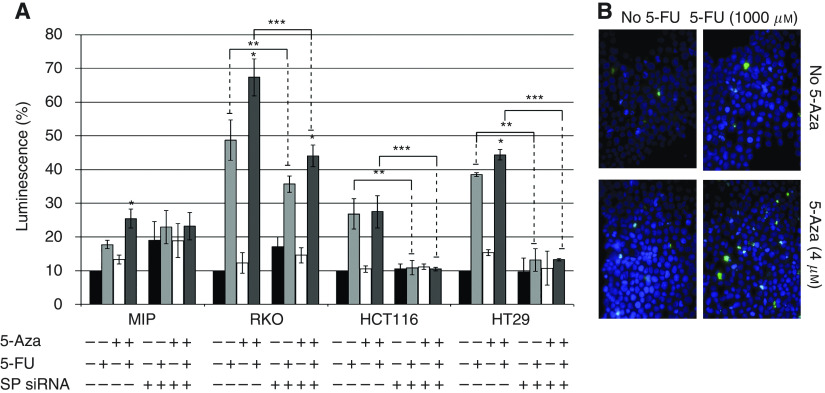
The effect of 5-Aza in combination with 5-FU on apoptosis was assessed by caspase 3/7 assay (**A**) and **B**) TUNEL assay (blue, DAPI nuclear stain; green, TUNEL-positive cells). (*P*<0.05 for ‘^*^’ when compared with 5-FU treatment; ‘^**^’ comparison between 5-FU only vs SPARC siRNA(+)/5-FU(+) treated cells; and ‘^***^’ comparison between 5-Aza(+)/5-FU(+) and 5-Aza(+)/5-FU(+)/SPARC siRNA(+)). Results represented as mean±s.e.

The decrease in cell viability following incubation with 5-Aza and 5-FU exposure can be attributed to an increase in apoptosis, as significantly higher levels of caspase 3/7 were detected in MIP101, RKO and HT29 cells exposed to the 5-Aza and 5-FU combination, in comparison to 5-FU alone (Figure 5A). In MIP101 cells, caspase 3/7 levels increased from 17.7±1.3% (5-FU alone) to 25.4±2.8% (5-Aza+5-FU), *P*=0.03; while in RKO and HT29 cells, levels increased from 48.7±6.0% (5-FU alone) to 67.4±5.5% (5-Aza+5-FU), *P*=0.04 and 38.5±1.5% (5-FU alone) to 44.3±3.3% (5-Aza+5-FU), *P*=0.01 respectively. The effect of 5-Aza in augmenting caspase 3/7 levels, and hence, apoptosis, was again eliminated when *SPARC* gene expression was diminished following transfection of cells with SPARC siRNA (Figure 5A). In fact, reduction of SPARC by siRNA in all four cell lines dramatically reduced the response to 5-FU, even in the presence of 5-Aza. These observations were also confirmed by demonstrating significantly greater numbers of TUNEL-positive cells in MIP101 cells incubated with 5-Aza and 5-FU under similar conditions (Figure 5B).

### Cell proliferation decreases in colorectal cancer cell lines following incubation with 5-Aza-2′deoxycytidine

**Figure 6:**
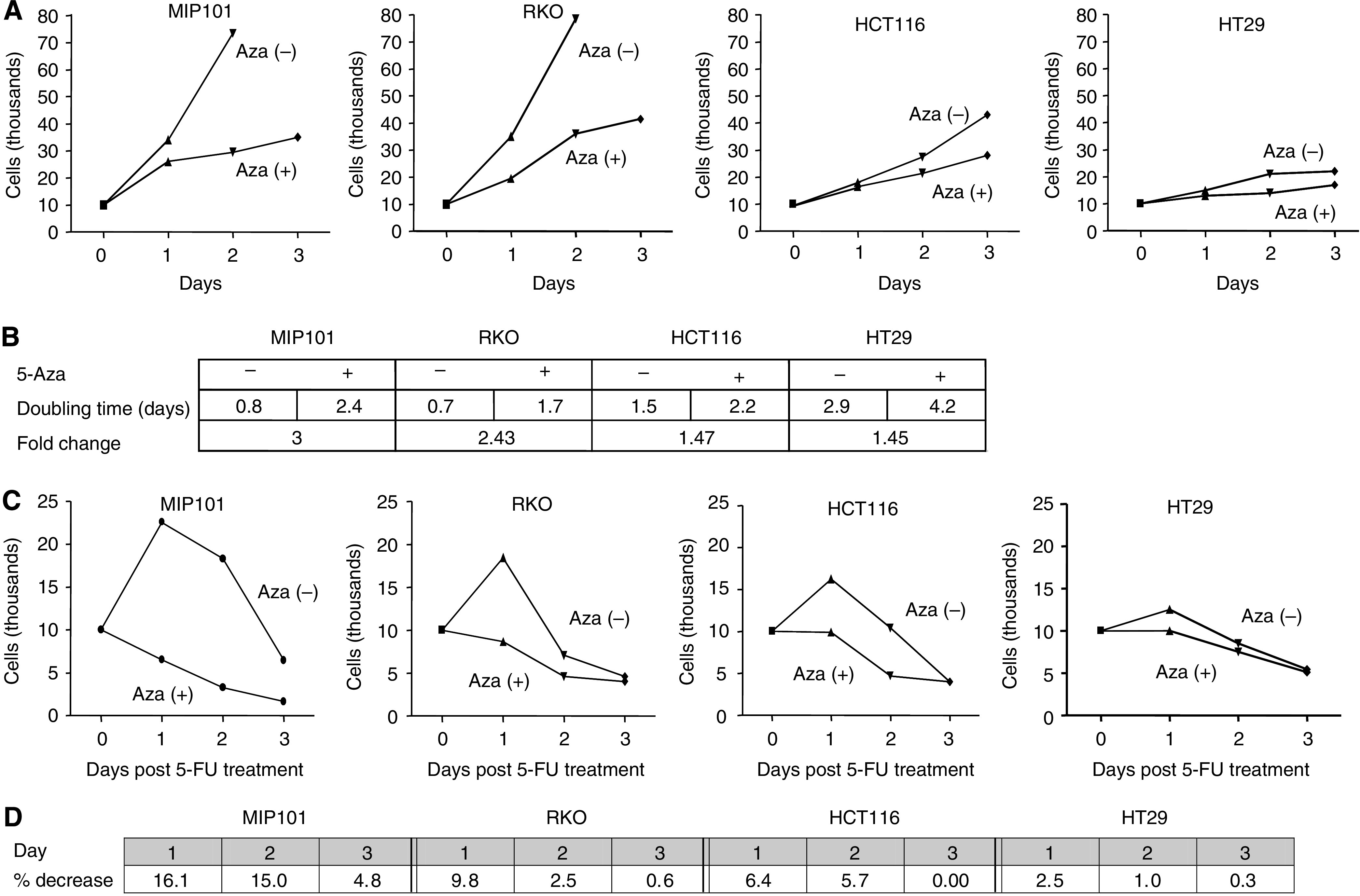
The effect of exposure of CRC cell lines to 5-Aza on cell proliferation. (**A**) Exposure to 5-Aza affected the cell proliferation in all four cell lines (MIP101, RKO, HCT 116, and HT29), with the greatest reduction in growth observed with MIP101 and RKO, resulting with a 3.0- and 2.4-fold increase in cell-doubling time (**B**). (**C**) In combination with 5-FU, cell proliferation decreased in all four CRC cell lines exposed to 5-Aza, with the greatest effect occurring within the first day of 5-FU treatment in MIP101 and RKO cells (**D**).

We next examined the effect of exposing our various CRC cells to 5-Aza on cell proliferation, as we had previously shown that, *in vitro*, higher levels of SPARC in cells were associated with delayed cell cycle progression (Tai *et al*, 2005). The most dramatic decrease in cell proliferation following incubation with 4 *μ*M 5-Aza was again noted with MIP101 and RKO cells, which have complete methylation of the SPARC promoter, beginning as early as 24 h of incubation (Figure 6A), with a 3- and 2.43-fold increase in doubling time (from 0.8 days to 2.4 days, and 0.7 to 1.7 days) in MIP101 and RKO, respectively (Figure 6B). In HCT 116 and HT29 cells with partial methylation of the SPARC promoter, a less dramatic yet significant decrease in cell proliferation was also observed when these cells were incubated with 5-Aza, which was most noticeable after 2 days (Figure 6A and B).

Exposure to 5-FU, in addition to 5-Aza, resulted in a steady decline in cell numbers in all cell lines, but most dramatically within 24 h, in both MIP101 and RKO cells in comparison to HCT116 and HT 29 cells (Figure 6C and D).

## DISCUSSION

We previously showed that *SPARC* gene and protein expression was decreased in colorectal cancers and that lower levels were associated with reduced sensitivity to chemotherapy (Tai *et al*, 2005). In this study, we proceeded to examine the methylation status of the *SPARC* promoter, as aberrant methylation is commonly observed in colorectal cancers (Toyota *et al*, 1999; Lee *et al*, 2004), and recent studies in other cancers have revealed aberrant hypermethylation of the *SPARC* promoter to be responsible for low levels of SPARC expression (Sato *et al*, 2003; Suzuki *et al*, 2005; DiMartino *et al*, 2006). We were able to identify specific CpG sites that were consistently methylated in colorectal cancers by DNA bisulfite sequencing. Moreover, we demonstrated that repressed *SPARC* expression in colorectal cancer cell lines resulting from aberrant hypermethylation, could be reversed following exposure to a demethylating agent, 5-Aza-2′deoxycytidine, which resulted in increased SPARC expression and enhanced sensitivity of colorectal cancer cells to 5-FU.

In colorectal cancer, the change in *SPARC* expression follows a similar pattern as in pancreatic cancer: higher levels of SPARC are observed in normal pancreatic ductal epithelial cells while it is absent in the majority of pancreatic cancers (Sato *et al*, 2003). This loss of *SPARC* gene expression was also associated with aberrant hypermethylation which could be reversed by 5-Aza-2′deoxycytidine (Sato *et al*, 2003). Aberrant hypermethylation of the *SPARC* promoter has also been observed in cancers of the lung (Suzuki *et al*, 2005), prostate (Wang *et al*, 2005), endometrium (Rodriguez-Jimenez *et al*, 2007) and leukaemia cell lines (DiMartino *et al*, 2006). Recent studies using genome-wide screening to identify genes that are targeted for aberrant methylation in tumours have also consistently shown SPARC expression to be inducible by 5-Aza-2′deoxycytidine, in pancreatic (Sato *et al*, 2003), cervical (Sova *et al*, 2006), and colorectal cancer cell lines (Yang *et al*, 2007). In this study, we found an overall methylation pattern of 60% in the *SPARC* promoter of primary colorectal cancer specimens, while only 28% of the CpG sites were partially methylated. Normal colon had an equal distribution of complete, partial or no methylation of the *SPARC* promoter. This variable distribution in methylation status in the normal colon is interesting. The mean age of our subjects with normal colon was 54.2 years-old, while those with colorectal cancer was 66.4 years-old, which may lead one to question if this observation may be due to age-related methylation. However, differences in the methylation status of the *SPARC* promoter between a tumour specimen (95% methylation) and its surrounding normal colonic epithelium (50% methylation) were noted within the same individual, which would argue against the possibility of an age-related phenomenon, and instead, indicates that *SPARC* promoter hypermethylation in tumours to be most likely related to a diseased state. It is possible that the presence of complete or partial methylation in some of these pathologically ‘normal’-appearing colonic epithelium may, in fact, represent an early epigenetic event in the adenoma–carcinoma sequence that will predispose these patients to developing colorectal cancers. Our studies suggest that methylation of specific CpG sites (1–2, 8–10, 16–18) had the highest frequency of hypermethylation in colorectal cancers, which may prove useful as potential diagnostic or predictive markers of this disease. This hypothesis will require a prospective study to determine if partial or complete methylation of the *SPARC* promoter in the ‘normal’ colon can be predictive of future development of colonic polyps and later, colorectal cancers.

The results of our DNA bisulfite sequencing of the *SPARC* promoter, spanning a region 1500 bp upstream and including exon1/intron1, allowed us to determine that the *SPARC* promoter hypermethylation could be identified in 80% of cases of human colorectal cancers (8 of 10 cancer). This finding differs from a recent study by Yang *et al* (2007), where only partial methylation (and no hypermethylation) was observed in their cases of colorectal cancers within a shorter 221 bp region of the *SPARC* promoter (Yang *et al*, 2007). One possible explanation to account for this difference between our studies may be in the processing of the clinical samples: all of the samples used in the current study were laser capture microdissected to minimise potential contamination of either normal colonic epithelium in our colorectal cancer specimens, and vice versa. In the study by Yang and colleagues, bulk tissues were used. Our results are also more in keeping with sequencing results of a similar region spanning exon1/intron1 of *SPARC* in human endometrial cancers, where 66% of the tumours were hypermethylated (Rodriguez-Jimenez *et al*, 2007).

We recognise that 5-Aza-2′deoxycytidine, a nucleoside antimetabolite and a potent inhibitor of DNA methyltransferase 1 (Dnmt1) activity, will have a global effect on other methylated genes (Soengas *et al*, 2001; Zhu *et al*, 2001; Scott *et al*, 2006) and does not specifically target SPARC. However, the results presented in this study demonstrate that exposure of CRC cell lines to 5-Aza effectively demethylated the CpG regions within the *SPARC* promoter, leading to greater *SPARC* gene and protein expression. We were also able to show that, in the absence of SPARC, the effect of 5-Aza in enhancing chemotherapy sensitivity could be abolished, thus indicating that, as a consequence of *SPARC* promoter demethylation by 5-Aza, higher SPARC levels led to greater chemosensitivity and improvements in chemotherapy response. Moreover, our results demonstrate that 5-Aza was most effective in those cell lines that either had complete hypermethylation of the *SPARC* promoter, such as MIP101 and RKO cells; or cells with a hemimethylated status, such as HT 29 cells, that responded to 5-Aza by completely demethylating the *SPARC* promoter. These observations suggest that cells with the greatest change in methylation status of the *SPARC* promoter in response to 5-Aza pretreatment, became the most sensitive to chemotherapy as a result of this increase in SPARC expression.

We previously showed that administration of exogenous SPARC, in combination with chemotherapy, to be highly efficacious in achieving tumour regression in animal xenografts (Tai *et al*, 2005), and our current observations lead us to believe that the use of a demethylating agent that restores SPARC expression can also be an effective adjunct to chemotherapy in colorectal cancer, and possibly other tumours with *SPARC* promoter hypermethylation. The clinical utility of 5-Aza has been restricted to the treatment of leukaemias and lymphomas (Karon *et al*, 1973; McCredie *et al*, 1973; Rivard *et al*, 1981), but no significant effect has been demonstrated with solid tumours (Moertel *et al*, 1972) because of two major reasons: (1) its short half life prevents adequate accumulation deep within solid tumours, and (2) high doses required to achieve clinical efficacy are associated with toxicity (Abele *et al*, 1987). However, our results suggest that a demethylating agent would function as a chemosensitiser and therefore, lower doses could be administered in combination with cytotoxic agents to achieve the desired clinical response.

To conclude, the findings of our current study demonstrate that: (1) *SPARC* is frequently aberrantly methylated in colorectal cancers, (2) specific methylated CpG sites have been identified that are present within the *SPARC* promoter of colorectal cancers, and (3) higher expression of SPARC can be achieved with 5-Aza-2′deoxycytidine, leading to improved sensitivity to chemotherapy. Together, these results provide a promising rationale to explore and translate the findings into further potential clinical and diagnostic therapeutic applications in colorectal cancer.

## Figures and Tables

**Table 1:** Demographics of patients with colorectal cancer or normal colon (clinical specimens used in this study)

**AJCC staging (colorectal cancer)**	**Subject age range (mean)**	**Gender (M/F)**
I	44–81 (64)	2/1
II	66–76 (71)	3/1
III	75 (75)	1/0
IV	55–58 (56.5)	2/0
Normal colon	34–68 (54.2)	3/2
